# Physiological and Transcriptomic Analysis of Bread Wheat MicroRNAs in Response to Zinc Availability

**DOI:** 10.3390/biom16010075

**Published:** 2026-01-02

**Authors:** Shuhan Sun, Yanlong He, Peng Chen, Cheng Chang, Lingyao Kong

**Affiliations:** College of Life Sciences, Qingdao University, Qingdao 266071, China; sunshuhan@qdu.edu.cn (S.S.); heyanlong@qdu.edu.cn (Y.H.); chenpeng1@qdu.edu.cn (P.C.); cc@qdu.edu.cn (C.C.)

**Keywords:** high-throughput sequencing, microRNAs, bread wheat, Zn deficiency, excess Zn

## Abstract

Zinc (Zn) is a mineral micronutrient that is essential for plant growth and development. Soil Zn deficiency or excess severely impacts plant health and crop yields. MicroRNAs (miRNAs) play crucial roles in plant responses to abiotic stress, but their roles in Zn homeostasis in important crop bread wheat (*Triticum aestivum* L.) remain unknown. This study investigated miRNA expression profiles in wheat roots under different Zn supply conditions using high-throughput sequencing. Phenotypic and physiological analyses revealed that high Zn promoted wheat plant growth, while low and excess Zn resulted in wheat plant growth inhibition and oxidative stress. A total of 798 miRNAs (including 70 known and 728 novel miRNAs) were identified; among them, 10 known and 122 novel miRNAs were differentially expressed. Many key miRNAs, such as miR397-5p, miR398, 4D_25791, and 5A_27668, are up-regulated under low Zn but down-regulated under high Zn and excess Zn. Target gene prediction and enrichment analysis revealed that the regulated genes of these miRNAs focused on “zinc ion transmembrane transporter activity”, “divalent inorganic cation transmembrane transporter activity”, and “cellular detoxification” processes in the low Zn vs. CK group. However, “glutathione metabolism” and “ABC transporter” pathways were obviously enriched in high Zn vs. excess Zn conditions, implying their potential functions in alleviating the oxidative damage and Zn efflux caused by Zn toxicity. Together, this study identified key miRNAs that respond to both Zn deficiency and excess Zn in bread wheat, revealing distinct regulatory patterns of the target genes in different Zn supply conditions. These findings provide a new field and valuable candidate miRNAs for molecular breeding aimed at improving zinc’s utilization efficiency in wheat.

## 1. Introduction

Zinc (Zn) is an important micronutrient for both plants and animals [1]. It is estimated that 5–6% of the prokaryotic proteome and approximately 9% of the eukaryotic proteome are zinc-binding proteins [2]. Maintaining Zn homeostasis in plants is of great significance for improving agriculture and human health. Zn deficiency represents a serious agricultural issue, severely limiting crop yield and nutritional quality. Conversely, excess Zn concentration results in cellular toxicity by binding to inappropriate ligands within cells and/or competing with other metal ions for enzyme or transporter active sites [3]. To cope with low soil zinc (Zn) availability and to mitigate the effects of Zn deficiency or excess, plants have evolved a range of biochemical and physiological adaptive mechanisms that enhance Zn acquisition and utilization while improving tolerance to Zn stress.

Membrane transporters play a central role in Zn homeostasis, which is necessary for uptake, compartmentalization, vascular loading, and delivery into organelles for utilization [4]. Three major metal transporter families, the ZIP (zinc-regulated transporters, iron-regulated transporter-like proteins) family, the CDF/MTP (cation diffusion facilitators/metal tolerance proteins) family, and the HMA (heavy metal ATPases) family have been identified as key players in Zn uptake and transport in plants [5]. In plants, several ZIP family members have been confirmed to respond to Zn deficiency. Fifteen, five, six, and five ZIP transporters were identified in Arabidopsis, rice, barley, and wheat, respectively [6,7,8,9,10,11]. The expression of *AtZIP1*, *AtZIP3*, *AtZIP4*, *AtZIP9*, and *AtIRT3* could be rapidly induced by Zn deficiency in Arabidopsis [12], suggesting their important roles in enhancing Zn uptake capacity during Zn deficiency conditions.

Cationic diffusion facilitators (CDFs), on the other hand, contribute to Zn tolerance by mediating Zn sequestration into the vacuole. In Arabidopsis, two tonoplast-localized CDFs, AtMTP1 and AtMTP3, facilitate Zn efflux from the symplast to vacuoles under high Zn conditions [13,14]. For HMA (heavy metal ATPases) family members, AtHMA2 and AtHMA4 serve in the translocation of Zn from root to shoot in Arabidopsis [15], and OsHMA2 and OsHMA3 were predicted to perform similar roles in rice [16].

Notably, these families exhibit distinct expression patterns, subcellular localization, and transport specificity. Some serve as Zn influx transporters, such as the ZIP family, which transport divalent cations from the extracellular space into the symplast [5,17,18]. In contrast, the HMA and CDF families typically act as efflux transporters, which transport cations out of the cytosol, either to the apoplast or into organelles such as vacuoles [18]. These characteristics determine the role of transporters in Zn uptake, roots-to-shoots transportation, sequestration, and distribution in plants [3]. Therefore, the intracellular Zn homeostasis represents a finely regulated system that warrants further in-depth investigation.

MicroRNAs (miRNAs) are a type of endogenous non-protein-coding small RNAs with 20–24 nt in length, which play an important role in regulating gene expression at the transcriptional or post-transcriptional levels [19,20]. They are involved in various biological processes, including biotic and abiotic stresses, organ development, and hormone signaling [21,22]. It is worth noting that a growing number of studies have demonstrated that miRNAs play a crucial role in the absorption and transport of multiple nutrients and heavy metals in plants, including nitrogen (N), potassium (K), phosphorus (P), calcium (Ca), iron (Fe), manganese (Mn), zinc (Zn), copper (Cu), aluminum (Al), and mercury (Hg) toxicity. According to the response to nutrients or metal stress, miRNAs can be divided into the following categories. miR160, miR169, miR170, miR172, and miR393 were known to respond to nitrogen (N) deficiency by altering root architecture and nodule development [23,24]. Pv-miR156, Pv-miR157, Pv-miR159, Pv-miR160, Pv-miR164, Pv-miR166, Pv-miR169, Pv-miR170, Pv-miR172, Pv-miR319, Pv-miR390, Pv-miR395, Pv-miR396, Pv-miR397, and Pv-miR398a/b/c, Pv-miR399, Pv-miR408, Pv-miR2118, Pv-miR1511, Pv-miR1509 were considered to respond to manganese (Mn) stress [25]. MiR397a and miR398a/b/c were reported to regulate Zn/Cu-containing protein transcripts and contribute to Fe deficiency adaptation in *Arabidopsis thaliana* [26,27]. MiRNAs miR319, miR390, and miR393 were found to be associated with aluminum response [28], while Mt-miR160, Mt-miR162, MtmiR390, Mt-miR1507, Mt-miR1509, Mt-miR2086, Mt-miR2111, Mt-miR2634 play important roles in mercury (Hg) toxicity [29].

In recent years, many microRNAs have been identified in response to Zn deficiency in Arabidopsis, rice, and sorghum. Under Zn deficiency, miR398 expression was inhibited, while the expression of its target gene coding copper–zinc superoxide dismutase (CSD1/CSD2) increased, enhancing ROS clearance and adapting to Zn-deficient environments [27]. Zeng et al. identified 10 Zn-responsive miRNAs (including miR171g-5p, miR397b-5p, miR398a-5p, and miR171-5p) and their target genes by integrating analyses of miRNAome and transcriptome data in rice [30]. In sorghum, 19 Zn-responsive miRNAs were identified under Zn deficiency by microarray [31]. However, to our knowledge, there have been no reports on whether and how miRNAs regulate Zn deficiency and Zn toxicity in wheat.

In this study, we researched the molecular mechanisms of Zn deficiency and toxicity response in wheat seedlings by physiology and transcriptome analyses. A large number of differentially expressed miRNAs were identified in low Zn, high Zn, and excess Zn by high-throughput sequencing. Meanwhile, several putative targets of these miRNAs, which may play roles in Zn signaling, uptake, transport, or metabolism, were also identified. The results indicate that the expression of hundreds of miRNAs and thousands of protein-coding genes was altered under different Zn concentrations. Go and KEPP pathway analysis revealed significant enrichment in the ion transmembrane transporter, cellular detoxification, and MAPK signaling pathways. Therefore, this study provides a comprehensive overview of the miRNA-mediated regulatory network in response to Zn concentration variation in bread wheat.

## 2. Materials and Methods

### 2.1. Plant Materials and Growth Conditions

Hydroponic culture was conducted to investigate phenotypic differences in bread wheat (*Triticum aestivum* L.) cultivar Shannong 38 under different Zn treatment conditions. To synchronize germination, seeds were soaked in water at 4 °C for 3 days, followed by incubation at 22 °C for 5 days (usually, seeds started germinating on the second day). After removing the endosperm, seedlings were cultivated in Hoagland’s solution containing 1 mM Ca (NO_3_)_2_, 1 mM KNO_3_, 0.4 mM MgSO_4_, 0.2 mM NH_4_H_2_PO_4_, 20 μM Fe (III)-EDTA, 3 μM H_3_BO_3_, 1.0 μM (NH_4_)_6_Mo_7_O_24_, 0.5 μM MnCl_2_, 0.7 μM ZnSO_4_, and 0.2 μM CuSO_4_ for three weeks in the growth chamber under 16 h light (500 μmol m^−2^ s^−1^)/8 h dark with 50% relative humidity at 22 °C/18 °C.

### 2.2. Treatments and Experiment Design

In this study, we supplemented Hoagland’s solution with ZnSO_4_ to establish different zinc concentration treatments. Based on the literature [32] and our pre-experiment, four treatments were determined as follows: low zinc (0.005 μM ZnSO_4_), CK (normal Hoagland’s solution containing 0.77 μM ZnSO_4_), high zinc (50 μM ZnSO_4_), and excess zinc (500 μM ZnSO_4_). During the three-week treatment period, the growth conditions remained the same as those described above. The plant height, fresh weight, and dry weight were measured after three weeks.

### 2.3. Determination of Chlorophyll Content, MDA Content, and Peroxidation Content

The chlorophyll content was measured as described previously [33]. Briefly, 0.5 g fresh leaf samples were used to extract chlorophyll in 80% acetone, and chlorophyll a and b content represent the total chlorophyll content. Then, 665 nm and 649 nm were employed to measure the sample’s absorbance using a spectrophotometer (SHIMADZU, Japan). The contents of chlorophyll a and b were computed as follows:

Ca (mg/L) = 13.95 × A_665_ − 6.88 × A_649_

Cb (mg/L) = 24.96 × A_649_ − 7.32 × A_665_

Ca: the content of chlorophyll a; Cb: the content of chlorophyll b

The total chlorophyll content was computed as follows:

Chlorophyll content (mg/g) = [(Ca + Cb) × V × *n*]/m

V: extraction volume (L); *n*: dilution-multiple; and m: sample weight (g).

Malondialdehyde (MDA) content was examined as described previously [34]. The second fresh leaf samples from each seedling were ground in liquid nitrogen, and 9 mL of 50 mM PBS (pH 7.8) were added to form a homogenate. Then, the homogenates were centrifuged at 13,000× *g* for 15 min at 4 °C. The supernatants were used to determine the MDA content. Then, 1.5 mL of enzyme crude extract was mixed with 0.5 mL TBA (2-thiobarbituric acid) solution prepared in 5% trichloroacetic acid and boiled at 100 °C for 30 min. After cooling, the samples were centrifuged at 1000× *g* for 15 min. The absorbances were recorded at 450 nm, 532 nm, and 600 nm, respectively. The MDA content was calculated as follows:

MDA content = 6.45(OD_532_ − OD_600_) − 0.599OD_450_.

The peroxidation level was examined by 3,3′-diaminobenzidine (DAB) staining, as described previously [35]. The second leaf samples from the wheat seedlings were harvested and immersed in a 1 mg/mL DAB solution for 12 h. Then, the leaves were washed with 75% ethanol to remove excess dye and photographs were taken to visualize the peroxidation accumulation.

### 2.4. RNA Isolation, Library Preparation, and Sequencing

For each treatment, roots from at least five seedlings were collected as a single biological sample. The combined root samples were flash-frozen in liquid nitrogen and stored at −80 °C. The total RNA was extracted from the wheat root using TRIzol^®^ Reagent (Invitrogen, 15596026, USA), according to the manufacturer’s instructions. The RNA quality was assessed by the 5300 Bioanalyser (Agilent, USA) and quantified using the ND-2000 (NanoDrop Technologies, USA). Only a high-quality RNA sample (OD_260/280_ = 1.8~2.2, OD_260/230_ ≥ 2.0, RQN ≥ 6.5, 28S:18S ≥ 1.0, >1 μg) was used to construct the sequencing library. Small RNA libraries were prepared using the QIAseq miRNA Library Kit (Qiagen, 331505, Germany), following the manufacturer’s instructions, and mRNA libraries were generated by TruSeq^®^ Stranded Total RNA Library Prep (Illumina, 20022061, USA). Then, the sequencing library was performed on the NovaSeq X Plus platform at Majorbio Bio-Pharm Technology Co., Ltd. (Shanghai, China), using the NovaSeq Reagent Kit.

### 2.5. Sequence Analysis and Identification of Novel and Known miRNAs

Firstly, the raw reads were filtered to remove adapters, ambiguous residues, and low-quality reads. Then, all clean mapped tags were aligned with miRNAs in the miR Base 22.0 database (http://www.mirbase.org/) (accessed on 22 October 2025) to obtain the known miRNA. Next, the remaining reads were aligned with the Rfam database and Repbase database to remove ribosomal RNA (rRNA), transfer RNA (tRNA), small nuclear RNA (snRNA), small nucleolar RNA (snoRNA), and other ncRNA and repeats. Finally, the unannotated reads were predicted to be and identified as novel miRNAs using miRdeep2 software (v2.0.1.3) [36], based on the positions in the genome and their hairpin structures.

### 2.6. Prediction of Target RNAs of miRNAs

The PsRobot (v1.2) tool (http://omicslab.genetics.ac.cn/psRobot/) (accessed on 22 October 2025) was employed to predict the target genes for all the known and novel miRNAs that expressed differentially in different Zn treatment groups. The parameters in the prediction were set as the default from the web server [37].

### 2.7. Differentially Expressed mRNAs and miRNA Identification

The DESeq2 package in R software (v1.42.0) was applied to screen differentially expressed mRNAs (DEmRNAs) and miRNAs (DEmiRNAs). We identified the significantly differentially expressed miRNAs as those with *p* value < 0.05 and |Log2FC| ≥ 1. For mRNAs, the thresholds of |Log2FC| ≥ 1 and p-adjust < 0.05 were used to screen the significant DEmRNAs.

### 2.8. GO and KEGG Pathway Enrichment Analysis

Gene Ontology (GO) enrichment analysis of differentially expressed genes was performed using the Goatools (v1.5.2) (https://github.com/tanghaibao/GOatools) (accessed on 22 October 2025). GO terms with corrected p-adjust < 0.05 were considered to be significantly enriched by differentially expressed genes (DEGs) and differentially expressed miRNAs.

The Kyoto Encyclopedia of Genes and Genomes (KEGG) is a database resource (http://www.genome.jp/kegg/) (accessed on 22 October 2025) that integrates genomic, chemical, and systemic function information, aiming to analyze the functions of the biological systems through genome sequencing and other high-throughput experimental technologies, including signaling interaction networks and metabolic pathways. In this study, we used the Python scipy package (v2.7) to test the statistical enrichment of DEGs and DEmiRNAs target genes in KEGG pathways.

### 2.9. Validation of the Expression of miRNAs and Genes by qRT-PCR

qRT-PCR was employed to validate the high-throughput sequencing results. The reverse transcription and RT-PCR were performed using Mir-XTM miRNA qRT-PCR SYBR^®^ Kits (TakaRa, 638314, Japan). Genomic DNA residue was removed using DNase I (TakaRa, 2212, Japan). Reverse transcription was carried out with a 10 μL mixture, including 10 μg RNA, 5 μL mRQ buffer (2×), and 1.25 μL mRQ enzyme. We incubated the mixture at 37 °C for 1 h, then inactivated the reverse transcriptase at 100 °C for 5 min. The resulting cDNA was diluted to 100 μL and served as the template to check the miRNAs and gene expression. Real-time PCR was conducted in a 20 μL reaction volume with 10 μL SYBR Premix Ex Taq master mix (TakaRa, DRR041A, Japan), 4 μL of forward and reverse primer (0.2 mM), and 2 μL cDNA template with the program: 95 °C for 10 min, 40 cycles of 95 °C for 15 s, and 60 °C for 30 s. The 2^−ΔΔCT^ method was employed to calculate the relative expression, and the expression data were log2-transformed before analysis. U6 and Actin 1 were used as reference genes for miRNAs and genes expression analysis, respectively [24,38]. All the primers for qRT-PCR were listed in Supplementary Table S13.

## 3. Results

### 3.1. Morphological and Physiological Responses of Wheat to Different Zn Concentrations

**Figure 1:**
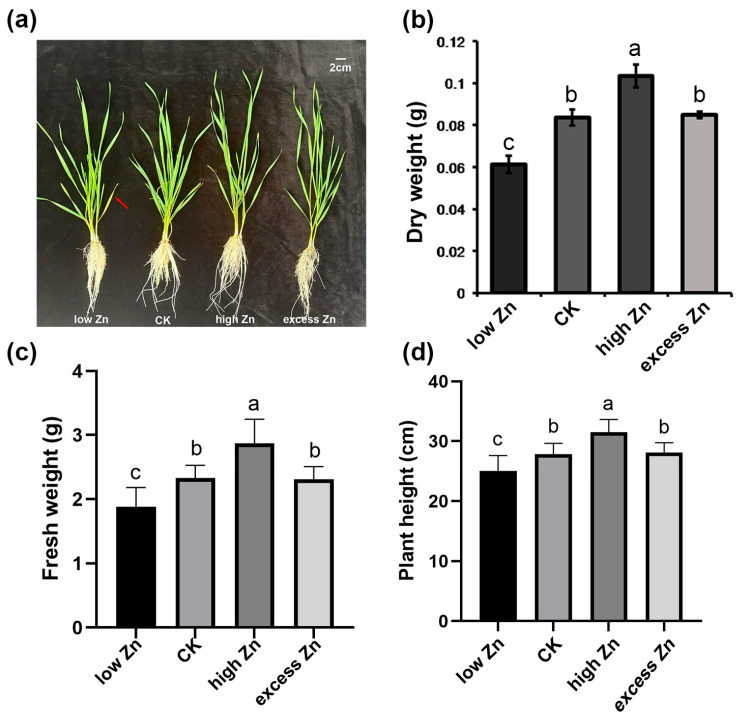
Morphological changes in wheat seedlings grown in a hydroponic solution with different Zn concentrations for three weeks. (**a**) The growth phenotype of wheat seedlings after three weeks. The red arrow indicated that the first leaf had yellowed. Scale bars = 2 cm. (**b**) Dry weight of each seedling after three weeks. (**c**) Fresh weight of each seedling after three weeks. (**d**) The plant height of each seedling after three weeks. Letters indicate a significant difference at the level of *p* < 0.05. Each treatment included at least nine seedlings. Three independent trials were performed with similar results in this figure.

To investigate the response of wheat to the Zn supply, wheat seedlings of the Shannong 38 variety were cultivated in a nutrient medium with three different Zn concentrations: low Zn (Zn deficiency), high Zn (optimal Zn), and excess Zn (Zn toxicity). Five-day-old wheat seedlings were transferred to hydroponic solutions with varying Zn concentrations, and plants were evaluated after three weeks of growth. As shown in Figure 1a, the wheat seedlings treated with high Zn (50 μM ZnSO_4_) exhibited optimal growth. In contrast, those subjected to low Zn (0.005 μM ZnSO_4_) and excess Zn (500 μM ZnSO_4_) showed obvious growth inhibition (Figure 1a). Meanwhile, those exposed to low Zn displayed yellowing of the first leaf after three weeks (Figure 1a). Then, biomass measurements further revealed that the high Zn treatment resulted in a significant increase in both the dry and fresh weights of the whole seedling (Figure 1b,c). The plant height also showed that high Zn treatment is significantly higher than the low Zn and excess Zn groups (Figure 1d).

**Figure 2:**
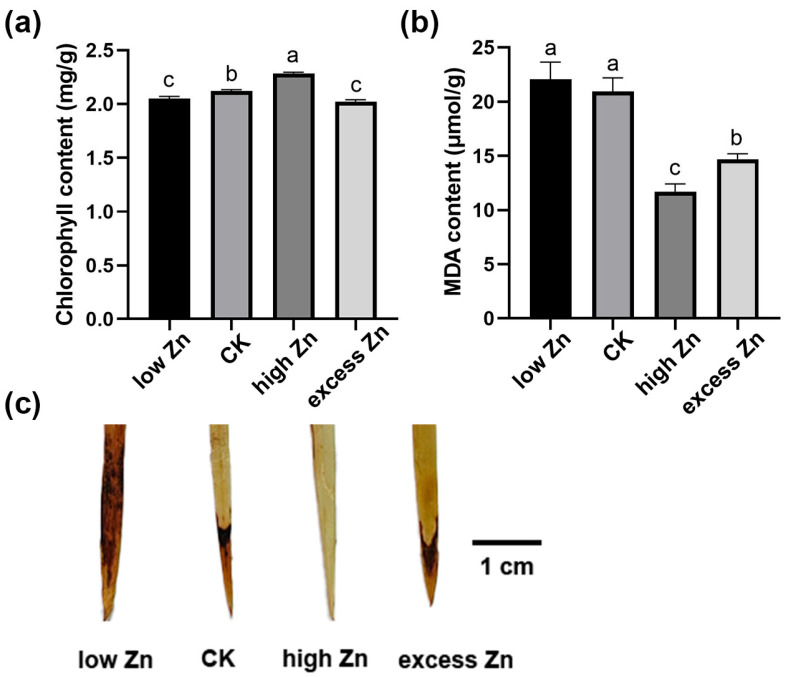
Physiological response of wheat seedlings to different Zn concentration treatments. (**a**) Chlorophyll content in leaves of wheat seedlings grown in different Zn concentration solutions for three weeks. (**b**) Malondialdehyde (MDA) content of wheat seedling leaves grown in different Zn concentration solutions for three weeks. (**c**) DAB staining of the wheat seedling leaves after three weeks of exposure to different Zn concentration solutions. Scale bars = 1 cm. Letters indicate a significant difference at the level of *p* < 0.05. Each treatment included at least nine seedlings. Three independent biological replicates were performed with similar results in this figure.

Then, the physiological changes in hydroponic solutions with different Zn concentrations were examined. First, chlorophyll content, an indicator of photosynthetic performance, was significantly higher in the high Zn group, whereas the low Zn treatment obviously reduced chlorophyll levels (Figure 2a), suggesting that low Zn may damage the photosynthetic system and inhibit the growth. Malondialdehyde (MDA) level is a marker of cytoplasmic membrane lipid peroxidation. Generally, the higher the MDA level, the greater cellular peroxidation and the more cellular damage [39]. Therefore, the MDA content was examined in different Zn concentrate treatments. As shown in Figure 2b, the high Zn group exhibited the lowest MDA content, followed by the excess Zn, CK, and low Zn groups, suggesting that an adequate Zn supply could reduce the malondialdehyde level and protect the lipid membranes from peroxidation damage.

To further investigate the accumulation of peroxides, the hydrogen peroxide (H_2_O_2_) level was examined by 3,3′-diaminobenzidine (DAB) staining. The intensity of DAB staining was distinctly higher in the low Zn group, indicating higher H_2_O_2_ accumulation and oxidative damage. In contrast, the DAB staining intensity was weaker in the high Zn group, reflecting lower H_2_O_2_ accumulation and oxidative damage (Figure 2c). Overall, these physiological findings align with the morphological phenotypes observed in Figure 1, suggesting the critical role of Zn homeostasis in promoting growth and minimizing oxidative stress in wheat.

### 3.2. Alterations in Transcriptome Profiles in Response to Zn Treatment in Wheat

To explore the transcriptional response of wheat seedlings to Zn treatment, seedlings were cultivated in different Zn solutions for three weeks. RNAs were collected from the roots for established libraries and RNA sequencing. A total of 12 samples were obtained under low Zn (0.005 μM ZnSO_4_), control (CK), high zinc (50 μM ZnSO_4_), and excess zinc (500 μM ZnSO_4_). Each treatment contains three independent biological replicates.

A total of 683.6 million raw reads were generated from the 12 transcriptome libraries by the NovaSeq X Plus platform. After filtering adaptors, junk, and low-quality sequences, 678.2 million clean reads were obtained.

Then, all clean reads were mapped to the wheat reference genome (accessed from the following: https://plants.ensembl.org/Triticum_aestivum/Info/Index, accessed on 22 October 2025) using HISAT2 software (2.2.1), and the percentage of clean reads in each sample ranged from 87.4% to 94.03% (Table 1).

### 3.3. Characterization of Small RNAs via High-Throughput Sequencing

**Figure 3:**
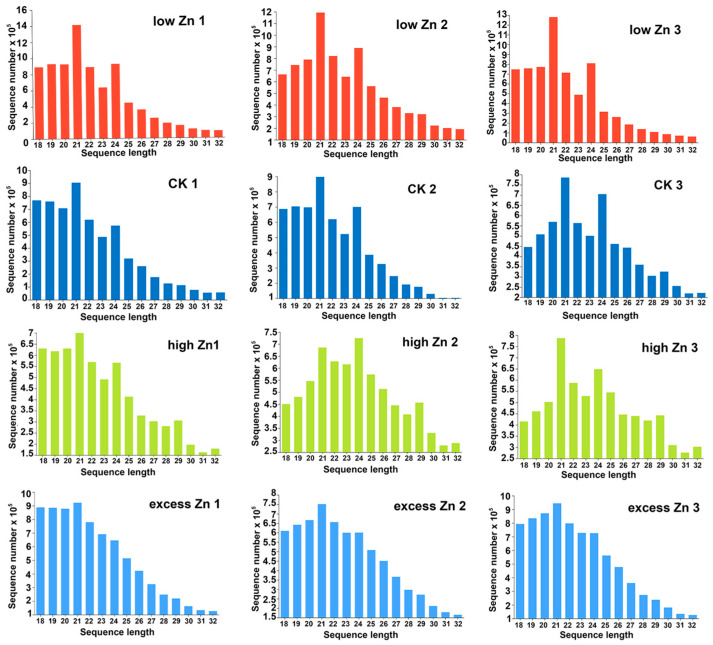
The length distribution of small RNAs in the twelve libraries. Low Zn 1, low Zn 2, and low Zn 3; CK1, CK2, and CK3; high Zn 1, high Zn 2, and high Zn 3; and excess Zn 1, excess Zn 2, and excess Zn 3 represent three independent libraries of low Zn, control, high Zn, and excess Zn treatment, respectively.

To identify the Zn response of miRNAs in wheat, 12 small RNA libraries were constructed synchronously and sequenced for the RNA samples mentioned above. Each library yielded approximately 10–12 million raw reads and 6.8–9.1 million clean reads after filtering (Table S1). Then, small RNAs ranging from 18 to 32 nt in length were subjected to further analysis. The length distribution of small RNAs was predominantly between 21 and 24 nt (Figure 3). However, there was a slight difference in the length profiles observed among different small RNA libraries. Specifically, in low Zn, CK, and high Zn libraries, 21 nt and 24 nt small RNAs accounted for higher proportions, whereas in the excess Zn libraries, 21 nt small RNAs were the most abundant (Figure 3).

### 3.4. Identification of Known miRNAs in Wheat

**Figure 4:**
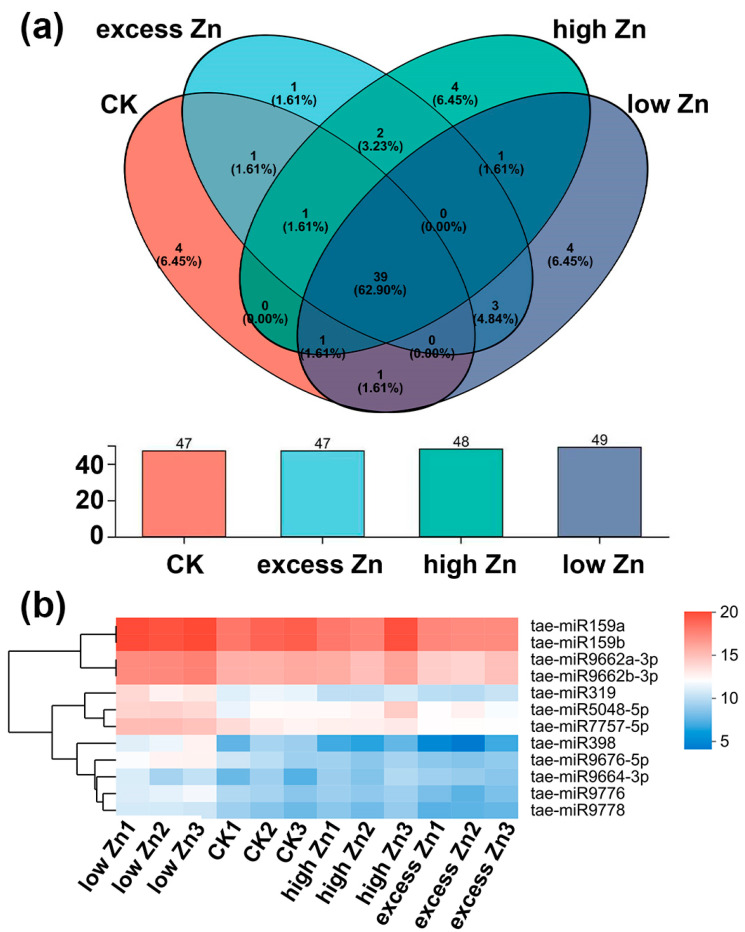
Venn diagram of known miRNAs identified in wheat with different Zn concentration treatments. (**a**) Known miRNAs in low Zn, CK, high Zn, and excess Zn, and the overlap between different groups. (**b**) The most abundant known miRNAs in four treatment conditions (reads number ≥ 1000; log2 transformed).

To identify known miRNAs from 12 libraries in this study, all clean reads were aligned against known *Triticum aestivum* miRNA precursors or mature miRNA sequences by miRbase 22.0 (http://www.mirbase.org/)(accessed on 22 October 2025). There were 49, 47, 48, and 47 miRNAs that matched with the known *Triticum aestivum* miRNA under low Zn, CK, high Zn, and excess Zn, respectively (Figure 4a). Finally, a total of 70 known miRNAs belonging to 27 miRNA families were identified across the 12 libraries, as summarized in Tables S2 and S3. The number of miRNAs within different miRNA families varies; miRNA1122, miRNA159, and miRNA9657 have the most members (three), followed by miRNA1120, miRNA167-1, miRNA395, miRNA444, miRNA9662, miRNA9666, miRNA9672, and miRNA9674, each with two members.

Then, we further investigated the distribution of the 70 known miRNAs among all the libraries. Of them, 39 miRNAs were detected in every library (Figure 4a). Additionally, four, four, four, and one miRNAs were specifically expressed in the low Zn, CK, high Zn, and excess Zn conditions, respectively. The expression levels of these miRNAs varied by a wide scope, with reads counting from a few to hundreds of thousands. Notably, 12 miRNAs exhibited relatively high expression levels, each with over 1000 reads in all 12 libraries. These highly expressed miRNAs belong to five families (Figure 4b; Table S3).

**Figure 5:**
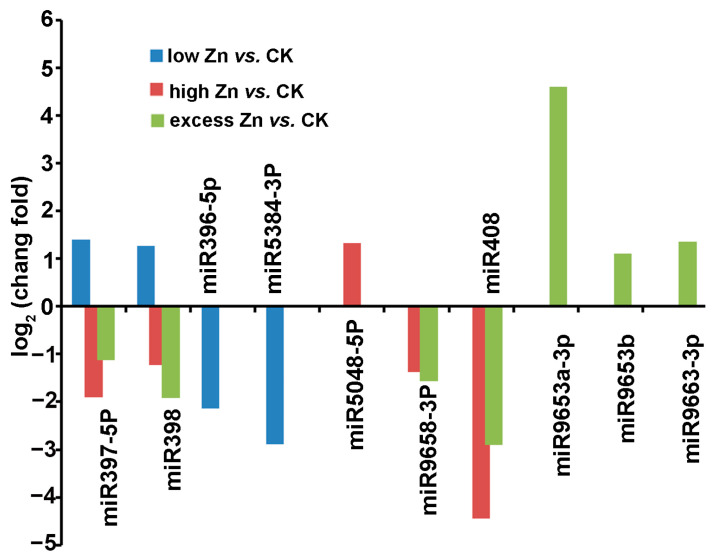
Differentially expressed known miRNAs were identified among different comparison groups. The relative changes in different Zn treatments were expressed as log2 ratios (low Zn vs. CK, high Zn vs. CK, and excess Zn vs. CK). A positive value means a higher miRNA expression in the different Zn treatment than in the CK, while a negative value indicates a lower miRNA expression.

Under different Zn concentrations treatments, 10 out of the 70 known miRNAs exhibited differential expression (|log2 fold-change| ≥ 1 and *p*-value ≤ 0.05) (Figure 5, Table S4). In the low Zn vs. CK comparison, miR397-5p and miR398 were up-regulated, while miR396-5p and miR5384-3p were down-regulated. In high Zn vs. CK, only miR5048-5p was up-regulated, whereas miR397-5p, miR398, miR9658-3p, and miR408 were down-regulated. In the excess Zn vs. CK group, miR9653a-3p, miR9653b, and miR9663-3p were up-regulated, while miR397-5p, miR398, miR408, and miR9658-3p were down-regulated. Noticeably, miR397-5p and miR398 were up-regulated in the low Zn vs. CK group, whereas they were down-regulated in the high Zn vs. CK and excess Zn vs. CK groups. Moreover, miR397-5p, miR398, miR408, and miR9658-3p shared consistent expression patterns (down-regulated) in both the high Zn vs. CK and excess Zn vs. CK comparisons (Figure 5, Table S4).

### 3.5. Identification of Novel miRNAs in Wheat

**Figure 6:**
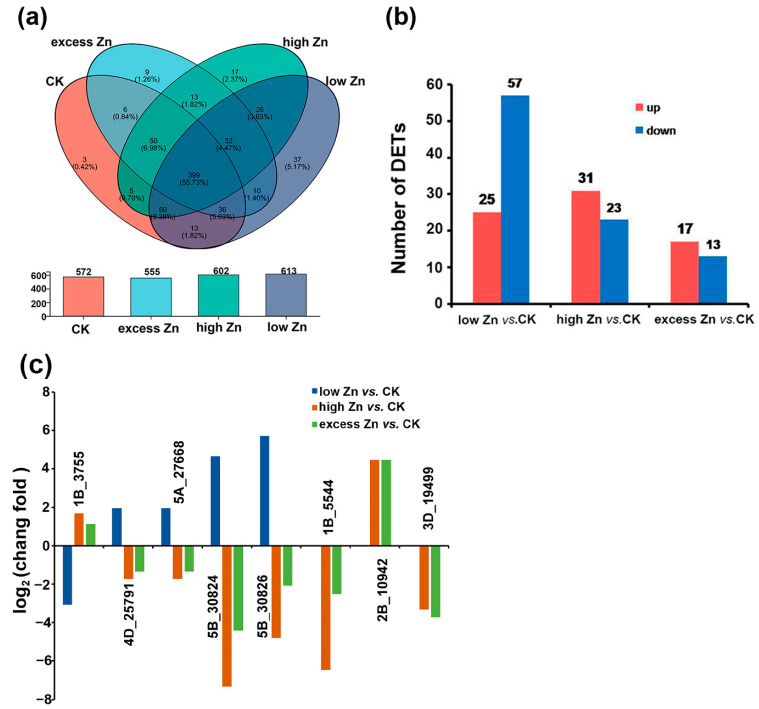
Novel miRNAs were identified in wheat with different Zn concentration treatments. (**a**) Venn diagram of novel miRNAs in different Zn concentration libraries and the overlap between different groups. (**b**) The number of up- or down-regulated novel miRNAs between different comparison groups (low Zn vs. CK, high Zn vs. CK, and excess Zn vs. CK). (**c**) Part of the differentially expressed novel miRNAs were identified among different comparison groups. The relative changes in different Zn treatments were expressed as log2 ratios (low Zn vs. CK, high Zn vs. CK, and excess Zn vs. CK). A positive value means a higher miRNA expression in the different Zn treatments than in the CK, while a negative value indicates a lower miRNA expression.

To identify putative novel miRNAs in wheat, novel miRNAs were predicted by miRDeep2. A total of 728 unique novel miRNAs were predicted in 12 small RNA libraries. Among them, 399 were detected in all the small RNA libraries (Figure 6a, Table S5). Additionally, 37, 3, 17, and 9 novel miRNAs were specifically expressed in the low Zn, CK, high Zn, and excess Zn conditions, respectively.

Differential expression analysis was also performed on the 728 novel miRNAs, revealing that 122 were differentially expressed (|log2 fold-change| ≥ 1 and *p*-value ≤ 0.05). In low Zn vs. CK, 82 novel miRNAs were differentially expressed, with 25 up-regulated and 57 down-regulated. For the high Zn vs. CK comparison, 31 novel miRNAs were up-regulated and 23 were down-regulated. Similarly, in the excess Zn vs. CK group, 17 were up-regulated, while 13 were down-regulated (Figure 6b, Table S6).

Interestingly, many of these differentially expressed novel miRNAs exhibited distinct expression patterns. For instance, 4D_25791, 5A_27668, 5B_30824, and 5B_30826 were up-regulated under low Zn but down-regulated under high Zn and excess Zn. In contrast, 1B_3755 was down-regulated under low Zn but up-regulated under high Zn and excess Zn. Similarly, several miRNAs displayed specific expression profiles: 1B_5544 and 3D_19499 were down-regulated in the high Zn and excess Zn groups but not detected in the low Zn treatment group, while 2B_10942 was up-regulated under high Zn and excess Zn (Figure 6c, Table S6).

### 3.6. Target Gene Prediction and Functional Classification of Zn-Response miRNAs

**Figure 7:**
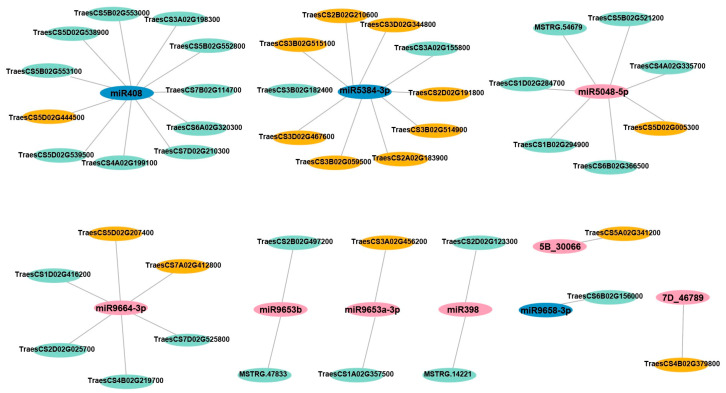
Network analysis between miRNAs and their potential Zn-responsive targets. The cytoscape platform was employed for analysis. Blue represents down-regulated miRNAs, while pink represents up-regulated miRNAs. Green represents down-regulated target genes, while orange represents up-regulated target genes.

The differentially expressed target genes of all the differentially expressed miRNAs identified in three comparison groups (low Zn vs. CK, high Zn vs. CK, and excess Zn vs. CK) were predicted by the psRobot (http://omicslab.genetics.ac.cn/psRobot/index.php) (accessed on 22 October 2025). In the low Zn vs. CK group, 13 targets were identified (Table S7), while 25 and 12 differential targets were identified in the high Zn vs. CK and excess Zn vs. CK comparisons (Tables S8 and S9), respectively. These differentially expressed miRNAs and differentially expressed target genes were shown in Figure 7.

The predicted target genes include stress-responsive genes, such as disease resistance protein (TraesCS2D02G025700, TraesCS7D02G525800, TraesCS2B02G497200, TraesCS7A02G412800). Several targets are involved in signaling transduction, including receptor kinase (TraesCS5D02G005300, TraesCS4A02G335700), receptor-like protein kinase (TraesCS1D02G284700, TraesCS1B02G294900), and protein kinase family proteins (TraesCS6B02G366500). Other identified target DEGs encode superoxide dismutase (TraesCS2D02G123300), xylanase inhibitor (TraesCS3B02G514900, TraesCS3D02G467600), acyltransferase (TraesCS3A02G198300), ubiquitin carboxyl-terminal hydrolase (TraesCS6B02G156000), methyl esterase (TraesCS3A02G456200), ammonium transporter (TraesCS3D02G344800), and Zinc finger AN1 domain-containing stress-associated protein 12 (TraesCS5D02G207400) (Tables S7–S9). However, most of these proteins have not yet been functionally characterized in wheat, and their roles in wheat growth and development remain largely unknown.

### 3.7. Prediction and Enrichment Analysis of miRNA Targets Responding to Low Zn

**Figure 8:**
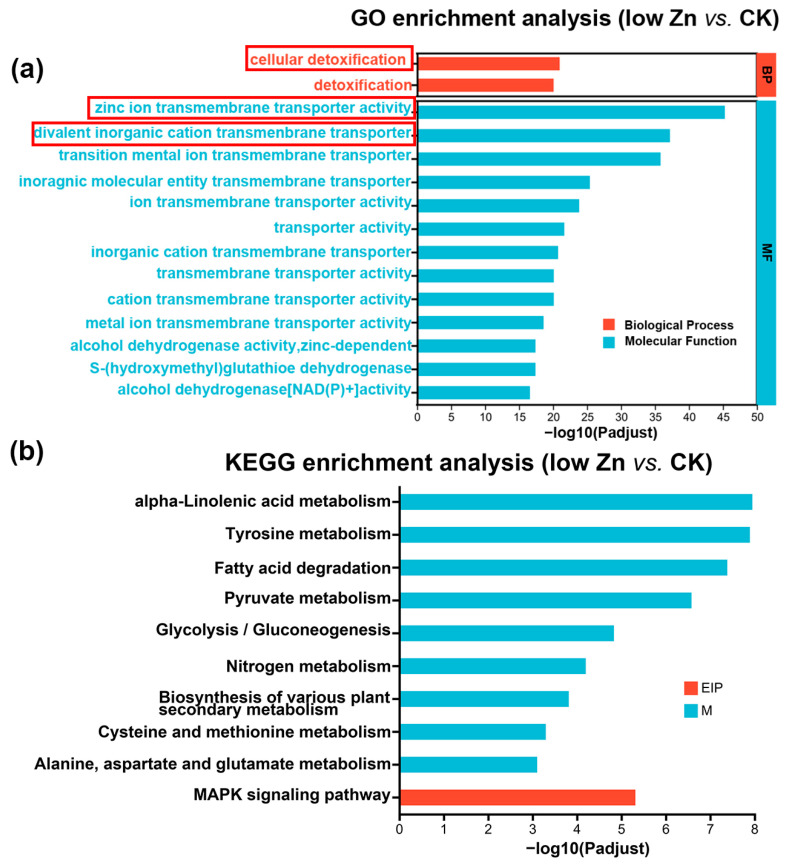
Comparison of Gene Ontology (GO) classification and the Kyoto Encyclopedia of Genes and Genomes (KEGG) pathway enrichment analysis of the target genes of differentially expressed miRNAs. (**a**) GO analysis of the target genes in low Zn vs. CK. The red box represents the significantly enriched GO terms. (**b**) KEGG enrichment analysis of the target genes in low Zn vs. CK. EIP and M mean environmental information processing and metabolism, respectively.

To elucidate the potential functional role of miRNA targets in response to low Zn conditions, we further performed Gene Ontology (GO) analysis using Goatools (https://github.com/tanghaibao/GOatools) (accessed on 22 October 2025). In the low Zn vs. CK group, a total of 554 GO terms were identified and classified into three major categories: 357 genes were assigned to biological process (BP), 13 to cellular component (CC), and 184 to molecular functions (MF) (Table S10). We employed the top 15 terms as the most significantly enriched GO terms. The results uncovered that under the low Zn vs. CK conditions, the differentially expressed genes were mainly concentrated in biological processes and molecular functions. Among these, the term “zinc ion transmembrane transporter activity” was the most significantly enriched, followed by “divalent inorganic cation transmembrane transporter activity” and “transition metal ion transmembrane transporter activity” terms. Meanwhile, the “cellular detoxification” term was also notably enriched (Figure 8a). These findings imply that zinc transmembrane transport and cellular detoxification processes are critically involved in maintaining essential physiological functions in low Zn conditions. In the same condition (low Zn vs. CK group), the Kyoto Encyclopedia of Genes and Genomes (KEGG) enrichment analysis further indicated significant enrichment in “alpha-linolenic acid metabolism”, “tyrosine metabolism”, and “MAPK signaling pathway” (Figure 8b), suggesting that these pathways may play important roles in low Zn stress in plants.

### 3.8. Prediction and Enrichment Analysis of miRNA Targets Responding to High and Excess Zn

**Figure 9:**
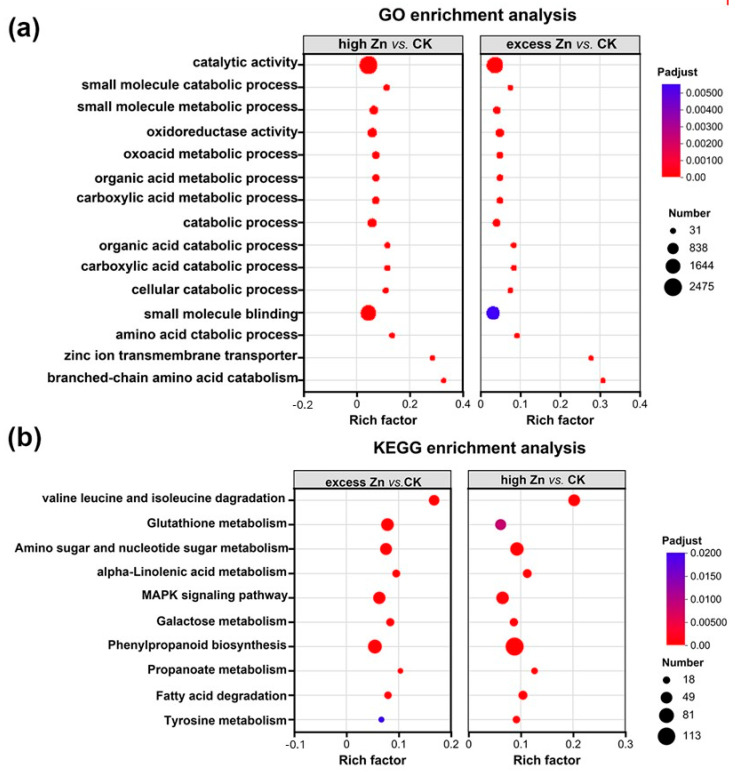
Comparison of GO classification and KEGG pathway enrichment analysis of the target genes of differentially expressed miRNAs. (**a**) GO analysis of the target genes in high Zn vs. CK and excess vs. CK. (**b**) KEGG enrichment analysis of the target genes in high Zn vs. CK and excess vs. CK.

In the comparative analyses of high Zn vs. CK and excess Zn vs. CK groups, GO and KEGG enrichment analysis were conducted as above. A total of 1672 GO terms were identified and categorized, with 956 genes assigned to biological process (BP), 100 to cellular component (CC), and 616 to molecular functions (MF) (Tables S11 and S12). Interestingly, the enriched GO terms in both the high Zn and excess Zn groups were highly similar to each other but distinct from those in the low Zn vs. CK group. Then, the top 15 terms were employed as the most significantly enriched GO terms. The results displayed that the “catalytic activity” and “small molecule binding” terms had more enrichment, while the “zinc ion transmembrane transport activity” term was less enriched (Figure 9a). KEGG enrichment analysis indicated similar results in both groups, with significant enrichment observed in “phenylpropanoid biosynthesis”, “glutathione metabolism”, and “MAPK signaling pathway” (Figure 9b), suggesting that these pathways play important roles in high and excess Zn conditions.

### 3.9. Differentially Enriched Analysis of miRNA Targets Responding Between High Zn and Excess Zn

**Figure 10:**
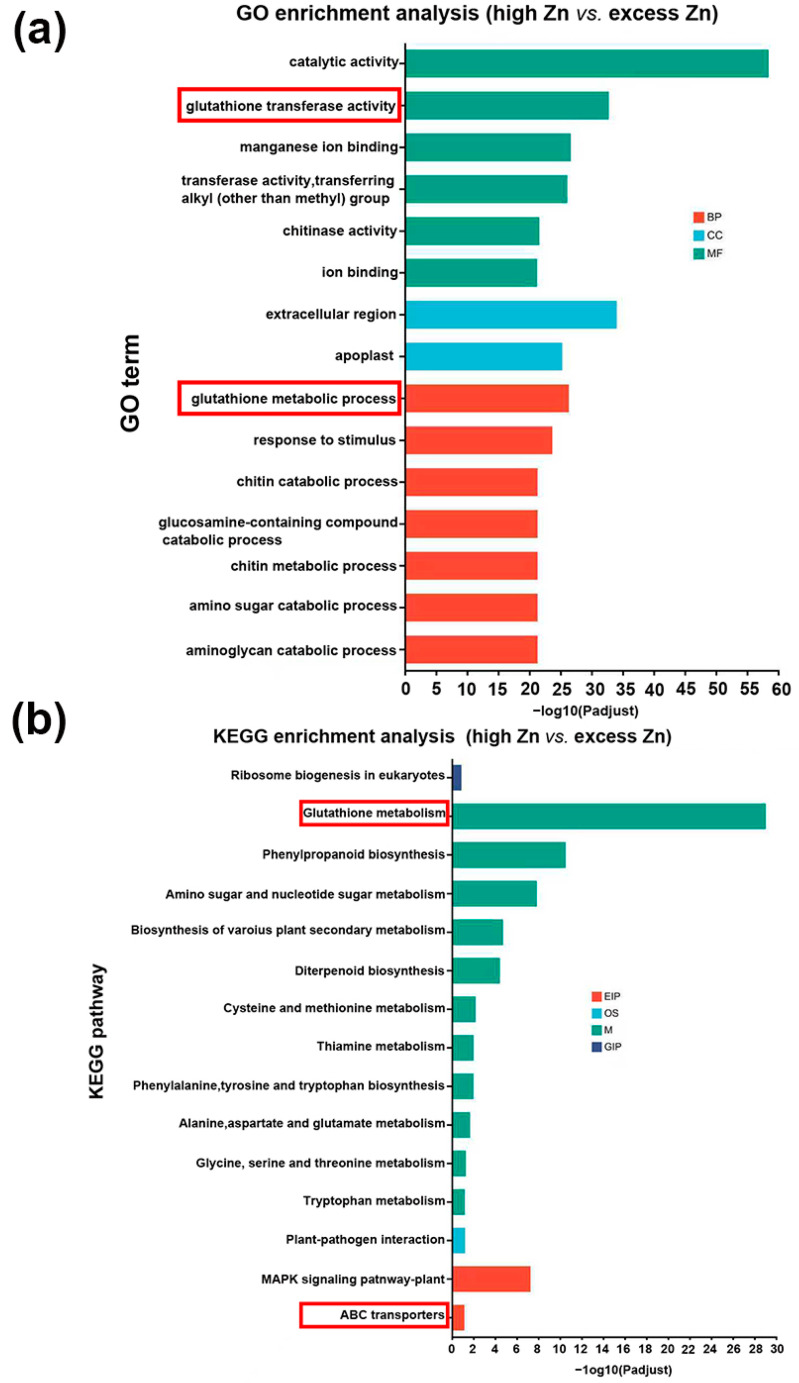
Comparison of Gene Ontology (GO) classification and Kyoto Encyclopedia of Genes and Genomes (KEGG) pathway enrichment analysis of the target genes of differentially expressed miRNAs. (**a**) GO analysis of the target genes in high Zn vs. excess Zn. The red box represents the significantly enriched GO terms. (**b**) KEGG enrichment analysis of the target genes in high Zn vs. excess Zn. The red box represents the significantly enriched KEGG pathway. EIP, OS, M, and GIP mean environmental information processing, organism system, metabolism, and genetic information processing, respectively.

As shown in Figure 1 and Figure 2, excess Zn application significantly inhibited the growth of seedlings. To further investigate the differences between high Zn and excess Zn supply, we performed GO and KEGG analysis to identify differentially enriched miRNA targets. Notably, GO terms related to “glutathione transferase activity” and “glutathione metabolic process” were significantly enriched (Figure 10a). Actually, glutathione is an important antioxidant that confers redox stability to the cell and influences plant responses to various environmental conditions [40]. Similarly, the KEGG pathway enrichment analysis revealed significant enrichment of “Glutathione metabolism”, suggesting that this process plays a major role in responding to excess Zn toxicity and relieving cellular oxidative damage. In addition, we found that the “ABC transporters” pathway was also enriched in excess Zn conditions compared with high Zn supply (Figure 10b). Previous studies have shown that ABC transporters are involved not only in cellular detoxification but also in regulating ion fluxes [41], suggesting their significant contribution to alleviating excess Zn toxicity in plant cells.

### 3.10. Validation of Differentially Expressed miRNA and mRNA by qRT-PCR

**Figure 11:**
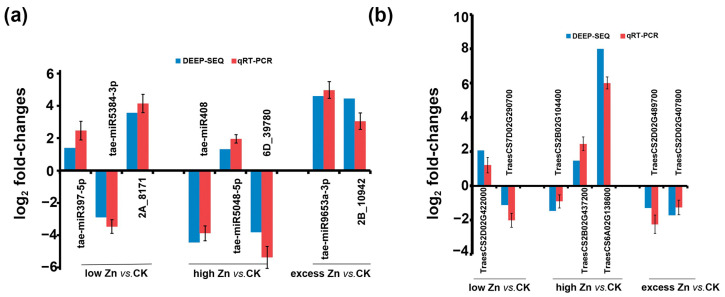
qRT-PCR validation of Zn-responsive miRNAs (**a**) and genes (**b**) in wheat. The 2^−ΔΔCt^ approach was used to calculate the fold changes in expression in qRT-PCR, with U6 and Actin 1 as reference for miRNAs and genes, respectively. All expression data were log2-transformed before analysis. The deep-sequencing results with log2 fold changes were shown to compare with qRT-PCR results.

Quantitative RT-PCR was carried out to verify the expression of miRNAs and genes. Five known miRNAs (tae-miRNA397-5P, tae-miRNA5384-3P, tae-miRNA408, tae-miRNA5-48-5P, and tae-miRNA9653a-3P) and three novel miRNAs (2A_8171, 6D_39780, and 2B_10942) were examined. The expression patterns of these miRNAs under low, high, and excess Zn cultivated conditions were consistent between the small RNA sequencing and qRT-PCR results (Figure 11a). Seven target genes were also selected for validation, including one gene encoding a zinc ion transporter (TraesCS2D02G422000), three involved in signaling pathways (TraesCS2B02G437200, TraesCS2B02G104400, and TraesCS2D02G489700), one associated with the peroxide clearance pathway (TraesCS7D02G290700), one involved in the auxin response pathway (TraesCS6A02G138600), and one encoding a heavy metal ATPase (TraesCS2D02G407800). The qRT-PCR results for selected genes were consistent with the RNA sequencing data (Figure 11b), confirming the high quality and reliability of the transcriptome datasets.

## 4. Discussion

**Figure 12:**
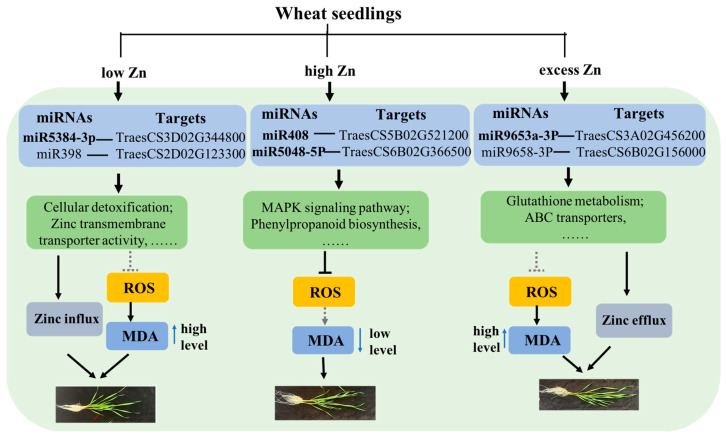
Proposed working model illustrating the differential response mechanisms of wheat under different Zn supply conditions. Bold miRNAs represent those that have been validated by qRT-PCR. The solid arrows indicate the promoting effects, while the dashed arrows indicate a weaker promoting effect. The solid “T” line suggests an inhibitory effect, while the dashed “T” lines denote a weaker inhibitory effect.

In this study, a low zinc supply significantly inhibited the growth of wheat seedlings, while high zinc levels promoted growth. However, an excessive zinc supply actually reduced the biomass accumulation to some extent (Figure 1). Physiological detection revealed a significant increase in chlorophyll content under high Zn conditions, along with a marked decrease in malondialdehyde (MDA) and peroxide levels (Figure 2), which is consistent with the observed growth phenotypes. Subsequently, high-throughput sequencing technology was performed. A total of 70 known and 728 novel miRNAs were identified to respond to varying zinc supplies. Compared with low Zn and CK, differentially expressed genes (DEGs) and target genes of differentially expressed miRNAs (DEmiRNAs) were mainly enriched in “zinc ion transmembrane transporter activity”, “divalent inorganic cation transmembrane transporter activity”, and “cellular detoxification” terms, suggesting that low Zn availability may enhance the expression of genes involved in zinc and divalent inorganic cation transport to sustain plant growth. Interestingly, under high and excess Zn conditions, multiple metabolic pathways were enriched, including “phenylpropanoid biosynthesis” and “glutathione metabolism”, indicating their important roles in sufficient Zn supply. However, compared with high Zn and excess Zn, “Glutathione metabolism”- and “ABC transporters”-related pathways were enriched, implying that these related genes and pathways may contribute to Zn efflux from intracellular to extracellular space under zinc excess. Overall, we propose a model for the differential responses of wheat seedlings under varying Zn supplies (Figure 12). This study enhances our understanding of miRNAs’ function in wheat in changing Zn supplies and provides a valuable set of candidate miRNAs that may serve as molecular markers for screening wheat germplasm with efficient zinc utilization.

### 4.1. miRNAs Play Significant Roles in Zn Uptake and Utilization

Zn serves as a component of more than 300 enzymes that are involved in the metabolic process of carbohydrates, lipids, proteins, nucleic acids, and other important biomolecules, thereby playing an important role in plant growth and development [3]. To maintain cellular Zn homeostasis under varying Zn conditions, plants have evolved sophisticated gene regulatory mechanisms. MicroRNAs (miRNAs) are non-coding small RNAs that are 21–24 nucleotide in length, serving as important modulators of gene expression at transcriptional and post-transcriptional levels in eukaryotes [42]. Recently, high-throughput sequencing studies have identified an enormous number of metal-responsive microRNAs in plants. To uncover the Zn-responsive miRNAs and elucidate mechanisms of Zn utilization in wheat, we performed whole transcriptome sequencing on the roots of bread wheat grown under different Zn concentrations.

After a strict bioinformatic analysis, a total of 798 miRNAs (including 70 known and 728 novel miRNAs) were identified in response to the Zn supply in wheat. Interestingly, several star miRNAs were discovered in our research. For instance, miR397-5p and miR396-5p were up-regulated in the low Zn group compared to the CK, but they were down-regulated in both the high Zn vs. CK and excess Zn vs. CK groups, indicating distinct expression patterns in response to Zn deficiency and high Zn levels. Moreover, miR397-5p, miR396-5p, miR408, and miR9658-3p shared similar expression trends, being down-regulated in both the high Zn vs. CK and excess Zn vs. CK groups.

In fact, miR397, miR396, and miR408 are known to participate in various developmental and physiological processes in plants. In Arabidopsis, miR397b targets the Casein Kinase II Subunit β 3 (CKB3), acting in the feedback loop of miR397-CKB3-CCA1 (CIRCADIAN CLOCK ASSOCIATED 1) to mediate the circadian rhythm and flowering [43]. In wheat, miR397 may target the transcription factor ICE1, which is involved in cold adaptation processes [44]. Multiple laccase (LAC) gene family members have been predicted as the targets of miR397, including AtLAC2, AtLAC4, and AtLAC7 in Arabidopsis [45], as well as OsLAC3, OsLAC6, OsLAC7, and OsLAC9 in rice [46]. In plants, LACs are involved in xylem lignin biosynthesis, maintaining the mechanical toughness of stems, and resisting lodging and adverse external stimulation [47]. Therefore, miR397 is involved in lignin biosynthesis and plant development by regulating LACs. Recent studies found that down-regulated miR397 lines showed more substantial Cd tolerance in Arabidopsis [47], suggesting a role for miRNA397 in heavy metal stress tolerance.

MiRNA396 is another of the most evolutionarily conserved miRNA families in eukaryotes. In Arabidopsis, miRNA396 post-transcriptionally repressed its target genes *GROWTH REGULATING FACTORS* (*GRFs*) to control seed size [48,49]. Overexpression of its target genes—*AtGRF1*, *AtGRF2*, and *AtGRF5*—leads to bigger seeds [50]. Similarly, in rice, miRNA396 represses its target genes, *OsGRF4* and *OsGRF*6, to mediate the seed size and grain yield [51,52]. A recent study further demonstrated that miRNA396 acts as a negative regulator of grain size and yield in soybean [53].

Another ancient and highly conserved microRNA is miRNA408, which is widely distributed in various plant species. MiR408 mediates the growth and development of different plants by down-regulating its targets, encoding blue copper (Cu) protein, and transporting Cu to plastocyanin (PC), thereby affecting photosynthesis and ultimately controlling yield-related traits and increasing the grain yield [54,55]. Additionally, miR408 improves tolerance to nutrient deficiencies and biotic and abiotic stresses and promotes the cellular antioxidant capacity by down-regulating target genes [55]. MiR408 also mediates the iron and Zn deficiency response in complex ways. For instance, under Fe deficiency in *Arabidopsis*, miR408 was up-regulated, while its target genes *LAC3*, *LAC12*, and *LAC13* were down-regulated [56]. In *Sorghum bicolor* leaves, miR408 was obviously induced in Zn deficiency, while the expression of its target gene, *Plantacyanin*, was decreased [31].

Although the biological function of miRNA397, miRNA396, and miR408 was characterized in Arabidopsis, rice, soybean, and *Sorghum bicolor*, their role in wheat under varying Zn supplies remains unclear. Our findings indicated that multiple miRNAs, represented by miRNA397, miRNA396, and miR408, are important in the wheat Zn response network, providing valuable genetic resources for breeding wheat varieties with improved Zn utilization efficiency.

### 4.2. The Crucial Role of the ZIP Family in Zn Homeostasis in Plants

Zn homeostasis is precisely regulated by various Zn transporter proteins. Among them, the Zinc/iron-regulated transporter-like proteins (ZIPs) have been widely reported to contribute to Zn homeostasis in previous studies in multiple species, including bread wheat [11]. In our study, Zn transmembrane transporter-related terms were obviously enriched in the low Zn vs. CK group in GO analysis (Figure 8). Specifically, several TaZIP genes were up-regulated under low Zn conditions in this study, including *TaZIP1*, *TaZIP2*, *TaZIP3*, *TaZIP4*, *TaZIP7*, *TaZIP10*, *TaZIP11*, and *TaZIP29*. These findings align with previous reports, supporting the reliability of our deep sequencing data. The ability of ZIP family members to respond to Zn deficiency and mediate Zn transport has been extensively reported. In Arabidopsis, AtZIP1 and AtZIP2 facilitate the translocation of Zn and Mn from roots to shoots [57]. In barley, the expression of six *HvZIP* genes is highly induced by zinc deficiency [10], suggesting their function in Zn uptake and root-to-shoot translocation under low Zn conditions. In rice, five OsZIPs (OsZIP1, OsZIP3, OsZIP4, OsZIP5, and OsZIP8) were involved in Zn transport, and three of them (OsZIP4, OsZIP5, and OsZIP8) specifically responded to Zn deficiency [10,58]. In hexaploid wheat, 14 ZIPs were identified, and 5 (TaZIP3, TaZIP6, TaZIP7, TaZIP9, and TaZIP13) were verified as Zn transporters [10]. In Tartary buckwheat (*Fagopyrum tataricum*), 13 ZIPs were identified, and 3 of them (FtZIP7, FtZIP10, and FtZIP12) transport Zn, 6 of them (FtZIP5, FtZIP6, FtZIP7, FtZIP9, FtZIP10, and FtZIP11) transport Fe, and 4 of them (FtZIP2, FtZIP3, FtZIP4, and FtZIP7) transport Cd [59], suggesting the multiple functions of ZIPs.

Consistent with these findings, our results show significant enrichment in the “zinc transmembrane transporter”, “divalent inorganic cation transmembrane transporter”, and “transition metal ion transmembrane transporter” terms. This supports the hypothesis that plants enhance Zn transport efficiency under deficiency to sustain relatively normal physiological processes.

### 4.3. The Function of Glutathione and ABC Transporter in Zn Tolerance in Plants

Glutathione (GSH) is the most important non-enzymatic antioxidant and detoxification in cells, playing a central role in relieving heavy metal toxicity. The detoxification process mainly includes the following aspects.

Reduced glutathione (GSH) can directly scavenge O^2−^ (superoxide anions), H_2_O_2_ (hydrogen peroxide), and singlet O_2_ [60,61]. On the other hand, under the action of glutathione peroxidase, GSH is oxidized to glutathione disulfide (GSSG), while the peroxides (such as H_2_O_2_ and lipid peroxides) are reduced to harmless water or alcohol, thereby protecting cell membranes from oxidative damage [62]. Therefore, the biosynthesis and metabolism of glutathione are important components of the stress response network in plants to resist oxidative damage and maintain the intracellular redox environment [63,64], serving as the first line of defense against heavy-metal-induced oxidative damage.

On the other hand, GSH serves as the direct substrate for phytochelation (PCs) biosynthesis, which plays a more critical role in heavy metal detoxification in plants, fungi, and algae. PCs bind with heavy metal ions to form PC-metal complexes [65,66]. Then, these complexes are transported to vacuoles or vacuole-like structures for storage under the mediation of ABC transporters [66,67], thereby separating heavy metals from sensitive areas such as the cytoplasm and achieving permanent detoxification.

The ABC transporter superfamily is one of the largest protein families, with hydrolyzed ATP to drive the transmembrane transport of molecules against concentration gradients [41]. Thus, ABC transporters are a class of key molecules for heavy metal detoxification. For instance, AtABCC1, AtABCC2, and AtABCC3 are the main transport proteins responsible for transporting PC-Cd complexes on the vacuolar membrane in Arabidopsis. The double mutant atabcc1 atabcc2 is extremely sensitive to cadmium, and the cadmium accumulation is significantly reduced in vacuoles [67,68].

In our study, the terms “glutathione transferase activity”, “glutathione metabolic process”, and “glutathione metabolism” pathway were obviously enriched in high Zn vs. excess Zn, suggesting Zn hyperaccumulation may elevate the glutathione levels to migrate and reduce the oxidative damage under excess Zn stress. Meanwhile, we also found that the ABC transporter pathway was enriched in the excess Zn group, suggesting a potential role in mitigating Zn toxicity by facilitating Zn efflux from the cytoplasm. However, the precise molecular function of the glutathione and ABC transporter still requires further exploration under excess Zn stress.

## 5. Conclusions

In this study, we found that high Zn (50 μM ZnSO_4_) was the optimal wheat growth Zn concentration, while both low (0.005 μM ZnSO_4_) and excess (500 μM ZnSO_4_) of Zn led to significant growth inhibition, chlorophyll reduction, and oxidative damage. miRNAs-mediated regulatory networks play a crucial role in response to the Zn concentration change in wheat. A total of 798 miRNAs were identified (70 known and 728 novel): among them, the key players, miR397-5p, miR398, 4D_25791, and 5A_27668, showed distinct expression patterns. They were up-regulated by low Zn and down-regulated by excess Zn. GO and KEGG enrichment analysis revealed that the “zinc ion transmembrane transporter activity”, “divalent inorganic cation transmembrane transporter activity”, and “cellular detoxification” terms were enriched in low Zn conditions, whereas the “glutathione metabolism” and “ABC transporter” pathways were obviously enriched in excess conditions, implying their roles in facilitating cellular oxidative damage. This study provides a valuable set of Zn-responsive miRNAs and their target genes, which act as fundamental resources and potential molecular markers for breeding in wheat, aimed at improving Zn-use efficiency and Zn tolerance.

## Figures and Tables

**Table 1:** Statistical summary of RNA sequencing reads in the 12 samples (libraries) from wheat roots cultivated in low Zn, high Zn, and excess Zn solution for three weeks. The labels CK1, CK2, and CK3; low Zn 1, low Zn 2, and low Zn 3; high Zn 1, high Zn 2, and high Zn 3; and excess Zn 1, excess Zn 2, and excess Zn 3 represent three independent biological replicates for the control, low Zn, high Zn, and excess Zn treatment, respectively.

Sample	Raw Reads	Clean Reads	Total Mapped	Multiple Mapped	Unique Mapped	Mapping Percentage (%)
low Zn 1	58,186,780	57,750,704	51,836,541	3,670,449	48,166,092	89.76
low Zn 2	53,889,272	53,463,878	49,256,940	3,358,308	45,898,632	92.13
low Zn 3	54,905,412	54,488,640	50,365,672	3,427,094	46,938,578	92.43
CK 1	59,220,060	58,736,976	55,231,763	3,707,867	51,523,896	94.03
CK 2	58,160,146	57,702,512	53,827,814	3,519,179	50,308,635	93.29
CK 3	60,921,576	60,435,612	56,324,316	3,864,653	52,459,663	93.2
high Zn 1	56,079,222	55,635,414	51,765,283	3,585,458	48,179,825	93.04
high Zn 2	53,609,902	53,192,442	49,703,693	3,302,778	46,400,915	93.44
high Zn	63,064,846	62,571,948	57,702,552	4,150,294	53,552,258	92.22
excess Zn 1	58,352,298	57,903,976	50,606,464	3,249,235	47,357,229	87.4
excess Zn 2	54,337,636	53,912,330	48,109,408	3,276,943	44,832,465	89.24
excess Zn 3	52,864,122	52,416,984	48,214,552	3,054,900	45,159,652	91.98

## Data Availability

The complete transcriptome sequencing data have been submitted to the Sequence Read Archive under the BioProject accession number PRJNA1367320 with the BioSample accession numbers SAMN53337301, SAMN53337302, SAMN53337303, SAMN53337304, SAMN53337305, SAMN53337306, SAMN53337307, SAMN53337308, SAMN53337309, SAMN53337310, SAMN53337311, and SAMN53337312.

## Supplementary Materials

The following supporting information can be downloaded at: https://www.mdpi.com/article/10.3390/biom16010075/s1, Table S1: Summary of small RNA sequencing data; Table S2: Known miRNA details table; Table S3: The miRNA family information; Table S4: The results of known miRNA expression levels; Table S5: Novel prediction miRNs; Table S6: Different expressed miRNA; Table S7: Target predicted in low Zn vs CK group; Table S8: Target predicted in high Zn vs CK group; Table S9: Target predicted in excess Zn vs CK group; Table S10: GO enrichment in low Zn vs. CK group; Table S11: GO enrichment in high Zn vs. CK group; Table S12: GO enrichment in excess Zn vs. CK group; Table S13: The primers for qRT-PCR.
