# Supplementary material for: Physiological and Transcriptomic Analysis of Bread Wheat MicroRNAs in Response to Zinc Availability
Source: Biomolecules. 2026 Jan 2;16(1):75. doi: 10.3390/biom16010075 (PMC12838673; doi:10.3390/biom16010075)
Supplement: Supplementary file 1 [file biomolecules-16-00075-s001.zip › Table S1-Summary of small RNA sequencing data.pdf]

Supplementary Table 1. The summary of small RNA sequencing data in the twelve libraries.

Sample	Raw Reads	Raw Bases	Clean Reads	Clean Bases	Error rate(%)	Q20(%)	Q30(%)	GC content(%)	Useful reads(18-32nt)
low Zn 1	13075697	980677275	8527578	193364093	0.0121	99.33	95.45	55.92	8259061
low Zn 2	12388337	929125275	9091069	219520118	0.012	99.37	95.69	55.61	8402819
low Zn 3	11730481	879786075	6996755	158254705	0.0122	99.2	95.26	55.84	6789050
CK 1	11042625	828196875	6180600	138764603	0.0118	99.46	95.95	57.03	5997618
CK 2	10696569	802242675	6849031	159740688	0.012	99.35	95.58	56.3	6481975
CK 3	10188821	764161575	8006789	206975222	0.0119	99.41	95.89	56.43	6666414
high Zn 1	11076938	830770350	7319591	182337147	0.012	99.35	95.69	55.03	6371443
high Zn 2	11094577	832093275	8857290	230999243	0.0119	99.38	95.78	55.61	7421973
high Zn 3	10572634	792947550	8580619	224837976	0.0119	99.39	95.82	55.59	7107484
excess Zn 1	13123893	984291975	8348794	195501071	0.012	99.37	95.54	57.6	7842854
excess Zn 2	10963699	822277425	7707464	189033660	0.012	99.39	95.69	56.84	6974829
excess Zn 3	12565000	942375000	8502200	199654280	0.012	99.43	95.63	57.98	8043293
